# Male Circumcision Increases Risk for Females

**DOI:** 10.1371/journal.pmed.0030072

**Published:** 2006-01-31

**Authors:** Jonathan Sykes

**Affiliations:** **1**Independent Researcher

Auvert et al. argue that male circumcision provides a protective effect for males [ 1]. On the other hand, Chao et al. identified circumcision of the male partner as a risk factor for females [ 2]. Auvert et al. do not provide information on the overall effect of male circumcision on HIV transmission and infection [ 1]. Male circumcision may in fact worsen the epidemic. It is imperative, therefore, that further studies be conducted to determine the overall effect before implementing mass circumcision campaigns to control HIV infection.

## References

[pmed-0030072-b1] Auvert B, Taljaard D, Lagarde E, Sobngwi-Tambekou J, Sitta R (2005). Randomized, Controlled Intervention Trial of Male Circumcision for Reduction of HIV Infection Risk: The ANRS 1265 Trial. PLoS Med.

[pmed-0030072-b2] Chao A, Bulterys M, Musanganire F, Habimana P, Nawrocki P (1994). Risk factors associated with prevalent HIV-1 infection among pregnant women in Rwanda. National University of Rwanda-Johns Hopkins University AIDS Research Team. Int J Epidemiol.

